# Establishing a prognostic model based on five starvation-related long non-coding RNAs for clear cell renal cell carcinoma

**DOI:** 10.18632/aging.204816

**Published:** 2023-06-20

**Authors:** Zhou Yu, Guo Chen, Zhenwei Feng, Yang Li, Haitao Yu, Wei Shi, Xin Gou, Chunlin Zhang, Xiang Peng

**Affiliations:** 1 Department of Urology, The First Affiliated Hospital of Chongqing Medical University, Chongqing, China; 2 Department of Urology, Suining Central Hospital, Suining, Sichuan, China; 3 Chongqing Key Laboratory of Molecular Oncology and Epigenetics, Chongqing, China

**Keywords:** clear cell renal cell carcinoma, lncRNA, starvation, risk score model

## Abstract

Background: Starvation-induced tumor microenvironment significantly alters genetic profiles including long non-coding RNAs (lncRNAs), further regulating the malignant biological characteristics (invasion and migration) of clear cell renal cell carcinoma (ccRCC).

Methods: Transcriptome RNA-sequencing data of 539 ccRCC tumors and 72 normal tissues were acquired from the TCGA and paired clinical samples of 50 ccRCC patients. *In vitro* experiments, such as qPCR, migration and invasion assays were applied to reveal the clinical relevance of LINC-PINT, AC108449.2 and AC007637.1.

Results: 170 lncRNAs were verified as starvation-related lncRNAs (SR-LncRs), of which 25 lncRNAs were associated with overall survival in ccRCC patients. Furthermore, a starvation-related risk score model (SRSM) was built based on the expression levels of LINC-PINT, AC108449.2, AC009120.2, AC008702.2 and AC007637.1. ccRCC patients with high level of LINC-PINT expression were divided into high-risk group and led to higher mortality, but AC108449.2 and AC007637.1 were contrary. Analogously, LINC-PINT was highly expressed in ccRCC cell lines and tumor tissues, especially in patients with advanced stage, T-stage and M-stage, while AC108449.2 and AC007637.1 showed the opposite results. In addition, the increased levels of AC108449.2 and AC007637.1 were significantly correlated with grade. Silencing LINC-PINT reduced the invasion and migration characteristics of ccRCC cells. SiR-AC108449.2 and siR-AC007637.1 enhanced the ability of invasion and migration in ccRCC cells.

Conclusions: In this study, we find the clinical significance of LINC-PINT, AC108449.2 and AC007637.1 in predicting the prognosis of ccRCC patients and verify their correlation with various clinical parameters. These findings provide an advisable risk score model for ccRCC clinical decision-making.

## INTRODUCTION

The incidence of renal cell carcinoma (RCC) accounts for 2-3 % of adult malignant tumors in the world [1]. RCC ranks 6th and 9th in the most common malignant tumors in males and females respectively, and it is the third most common tumor in urology [2, 3]. According to GLOBOCAN 2020 global cancer data statistics, RCC ranks 14th in incidence and 15th in mortality among all malignancies worldwide [4]. Clear cell renal cell carcinoma (ccRCC) is the most common subtype of RCC, and accounts for about 85% of all renal malignancies [5]. The main biological characteristics of ccRCC include genomic aberration and tumor metabolic modification [6]. At present, chromosomal copy number abnormalities are a clear molecular mechanism of ccRCC, such as chromosome 3p deletion and VHL inactivation [7]. In addition, mutations at FLCN, PTEN, BAP1 and other sites can also lead to ccRCC [8, 9]. Unfortunately, because of the lack of reliable tumor diagnostic markers, about 33% of ccRCC patients have already developed distant metastases when first diagnosed [10]. Surgical excision combined with targeted therapy is the current mainstream choice [11], these advances have increased the median survival of advanced patients from less than 10 months in 2004 to 30 months in 2011. However, about 30% localized ccRCC patients will still occur recurrence or metastasis after surgical resection [12].

In addition, with the rapid development of genomics and proteomics, precise therapy which refers to the treatment of precise etiology based on gene detection has applied to the treatment of ccRCC [13]. Increasing numbers of targeted drugs and immune drugs have been used in the precise therapy of ccRCC. Over the past few decades, accurate diagnosis and treatments on the basis of tumor-specific biomarkers, including genes and proteins, have been significantly developed and are gradually being applied in clinical practice [10]. SNRPA1, for example, was overexpressed in ccRCC, and was significantly associated with immune cell infiltration and immune checkpoint inhibitory genes, which could be a novel biomarker to predict ccRCC prognosis and affect tumor immunity [14]. HHLA2 had been shown a negative effect on the prognosis of ccRCC when co-expressed with PD-L1. Therefore, HHLA2 could be used as a predictor of clinical prognosis and immune checkpoint therapy in ccRCC [15].

The swift growth of tumor cells leads to the maldistribution of blood vessels, which further causes nutritional deficiency [16]. Starving environment significantly altered the genetic and protein characteristics of the cells, affecting tumor progression [17]. In pancreatic cancer, starvation-induced the expression of ERK1 / 2 and JNK kinases, mediating sensitivity to ferroptosis [18]. In melanoma and breast cancer, starvation caused REV1 SUMOylation and p53-dependent sensitization, which in turn promoted apoptosis of cancer cells and alleviated cancer progression effectively [19]. Therefore, identifying significant genetic alterations under conditions of starvation could lead to the discovery of more potential targets attractively, then provide new strategies for ccRCC treatment.

Long non-coding RNAs (lncRNAs) are a class of non-coding RNA molecules longer than 200 nt in eukaryotes, playing an important role in a variety of biological processes such as tumorigenesis, metastasis and malignant progression [20–25]. Numerous studies have shown that lncRNAs are over-expressed in renal tumor tissues and represent an important role in early diagnosis and prognosis evaluation of ccRCC [10]. LncRNA PVT1 is highly expressed in ccRCC tissues, and confirmed that it can form a positive feedback loop with HIF2α, promoting the progression of ccRCC and leading to poor prognosis. Therefore, PVT1 can be used as a biomarker to evaluate the prognosis of ccRCC [26]. However, the impact of starvation-mediated lncRNA alterations on the diagnosis and prognostic assessment of ccRCC has been rarely reported and needs to be further discovered and given more attention.

In the present study, we are in an effort to set up a new prognostic risk model of ccRCC on the basis of starvation-related lncRNAs (SR-LncRs). Moreover, the clinical significance of the starvation-related risk scoring model (SRSM) is sustained in a single-center cohort.

## RESULTS

### Establishing the SRSM through survival-related SR-LncRs (sSR-LncRs)

**Figure 1:**
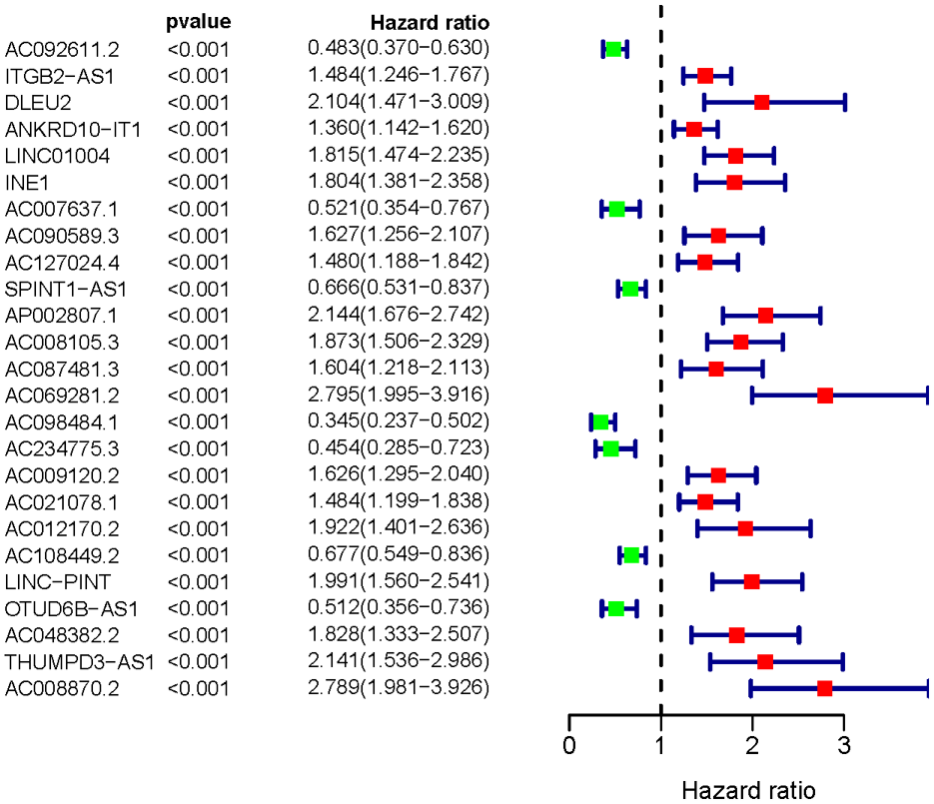
**The forest plot of sSR-LncRs.** The forest plot demonstrated the hazard ratios of AC108449.2, AC098484.1, AC092611.2, AC234775.3, OTUD6B-AS1, SPINT1-AS1 and AC007637.1.

**Figure 2:**
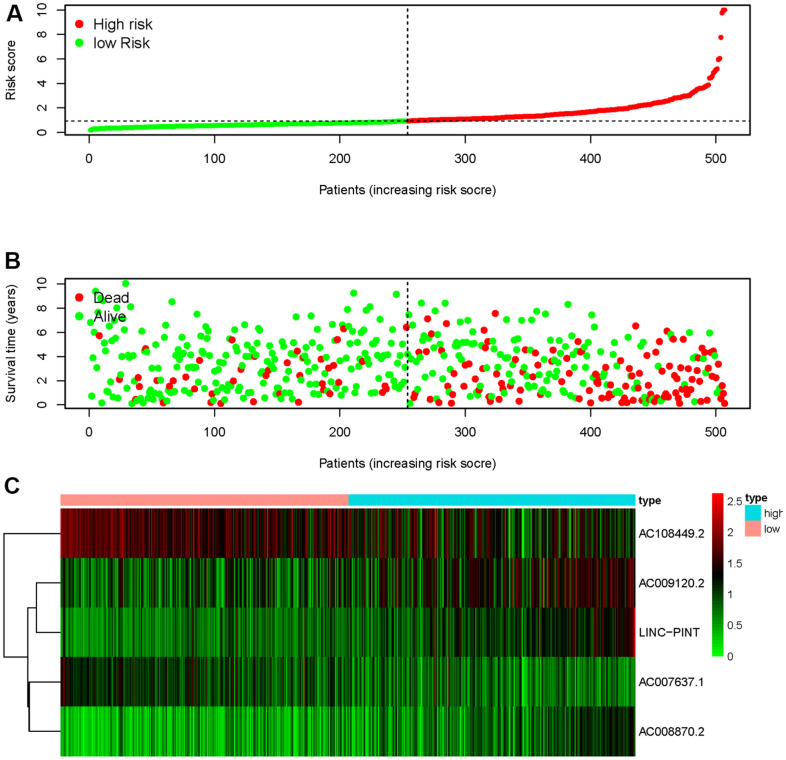
**Starvation-related risk score model.** The risk score of SRSM and the low-risk group was distributed on the left side by the median risk score (**A**). The survival status of ccRCC patients with the value of different risk scores (**B**). The heatmap of expressions of LINC-PINT, AC008870.2, AC108449.2, AC009120.2 and AC007637.1 in the SRSM (**C**).

**Figure 3:**
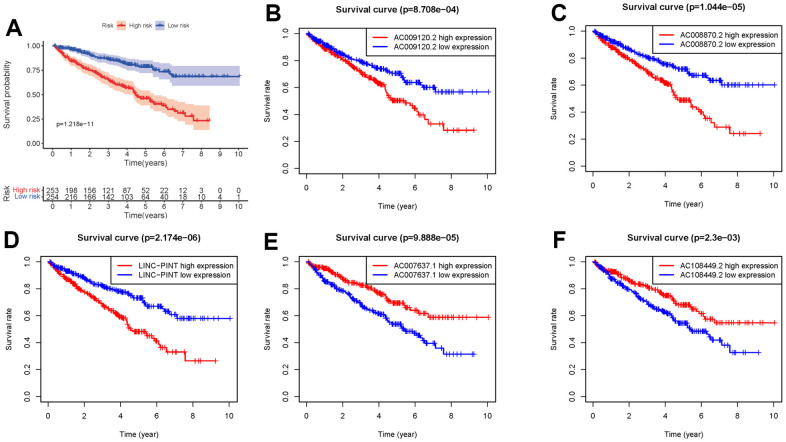
**Kaplan-Meier survival curve of SRSM.** Kaplan-Meier survival curve of the high-risk group and low-risk group in SRSM (**A**). The survival curves of sSR-LncRs (AC009120.2, AC008870.2, LINC-PINT, AC007637.1, and AC108449.2) (**B**–**F**).

We obtained the transcriptomic RNA sequencing data and the clinical data of ccRCC patients from the TCGA database. Subsequently, we verified the correlation between lncRNAs and 353 Starvation-related genes (SRGs) in M16522 and M41835 molecular characteristic database via Pearson correlation analysis. 170 lncRNAs were linked to starvation (| r | > 0.7 and P < 0.001). Next, we utilized univariate COX regression analysis to determine the correlation between SR-LncRs and the prognosis of ccRCC patients. As shown in the forest map, AC108449.2, AC098484.1, AC092611.2, AC234775.3, OTUD6B-AS1, SPINT1-AS1 and AC007637.1 are considered a protective element, but LINC-PINT, ANKRD10-IT1, ITGB2-AS1, AC021078.1, AC127024.4, AC009120.2, AC090589.3, AC087481.3, LINC01004, AC008105.3, INE1, AC048382.2, AC012170.2, AP002807.1, THUMPD3-AS1, DLEU2, AC069281.2 and AC008870.2 were detrimental parts (Figure 1). Then, we employed multivariate COX regression analysis in building SRSM. CcRCC patients were classified into high-risk and low-risk groups based on a median risk score (Figure 2A). Mortality and expression levels of LINC-PINT and AC008870.2 increased with increasing risk score, while AC108449.2, AC009120.2 and AC007637.1 decreased in patients with ccRCC (Figure 2B, 2C). The survival curve of SRSM showed that the high-risk group had the poor prognosis (Figure 3A). The high expressions of LINC-PINT and AC008870.2 were correlated with the poor prognosis. On the contrary, AC108449.2, AC009120.2 and AC007637.1 were related to favorable prognosis (Figure 3B–3F).

### Clinical relevance of sSR-LncRs

**Figure 4:**
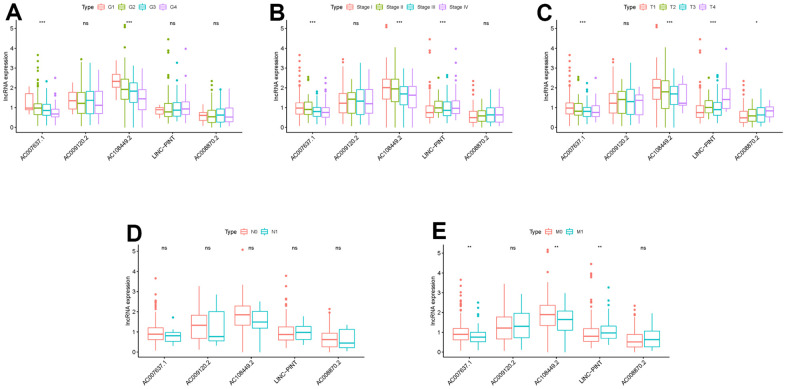
**Clinical correlation analysis of LINC-PINT, AC008870.2, AC108449.2, AC009120.2 and AC007637.1.** The relations between the expression levels of LINC-PINT, AC008870.2, AC108449.2, AC009120.2 and AC007637.1 with grade (**A**), stage (**B**), T-stage (**C**), N-stage (**D**) and M-stage (**E**).

**Figure 5:**
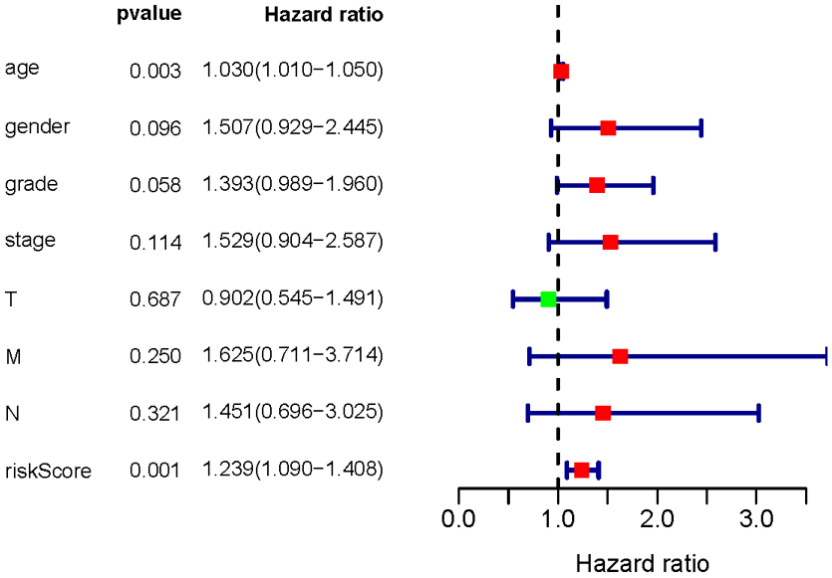
**Multivariate Cox analysis of SRSM.** Age and the risk score could be served as an independent prognosis factor of ccRCC patients.

**Figure 6:**
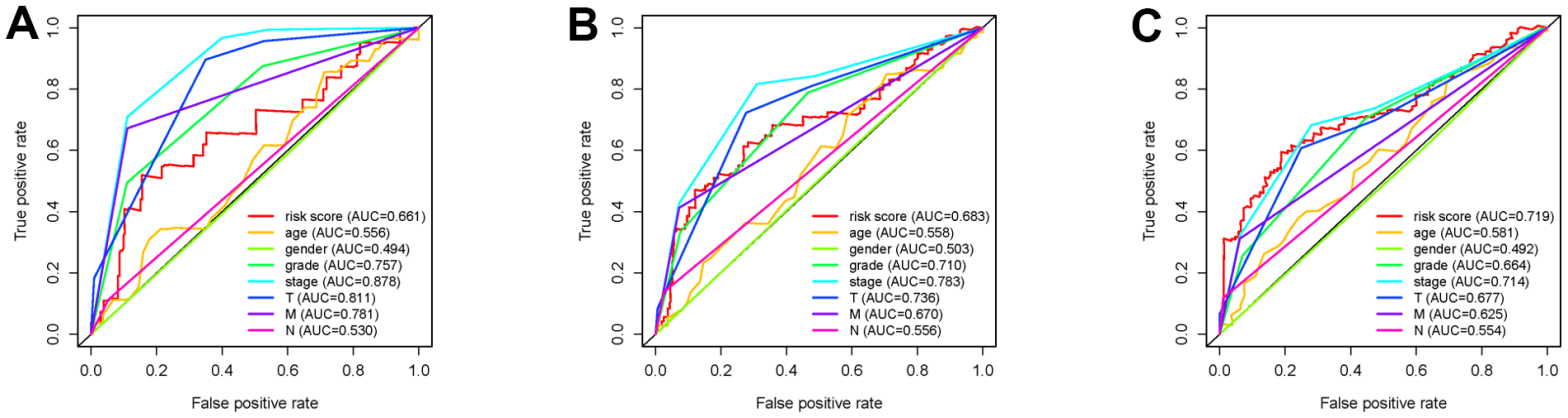
**Receiver operating characteristic (ROC) curves.** The area under curves (AUCs) of 1-,3- and 5-year SRSM and clinical characters. The 1-,3- and 5-year AUCs’ values of SRSM were 0.661 (**A**), 0683 (**B**) and 0.719 (**C**) respectively.

**Figure 7:**
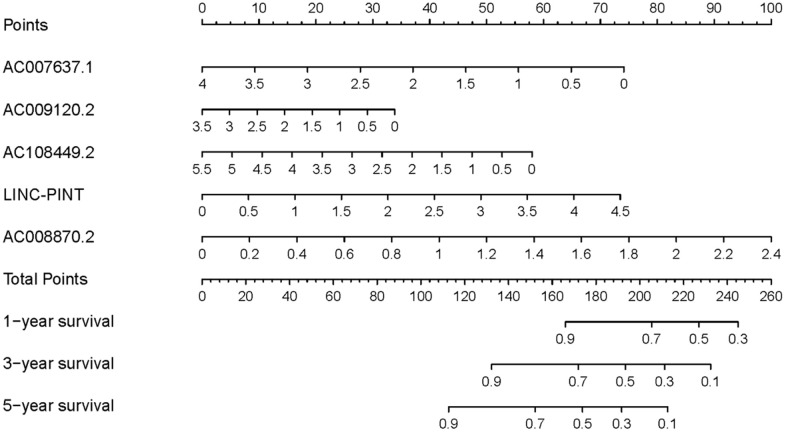
**Clinical underlying application of SRSM.** The nomogram of SRSM could predict 1-, 3- and 5-year survival probabilities of ccRCC patients by detecting the expressions of sSR-LncRs.

To investigate the association of sSR-LncRs with clinicopathologic features (e.g., grade, stage, TNM-stage) of ccRCC patients, we used the ggpubr package and discovered that LINC-PINT was highly expressed in ccRCC patients with advanced stage, T-stage and M-stage (Figure 4A–4E). However, the expressions of AC108449.2 and AC007637.1 were decreased in advanced grade, stage, T-stage and M-stage (Figure 4A–4E). Subsequently, we applied multivariate analysis to identify potential independent risk factors for ccRCC patients, and the results suggested that age and the risk score could be used as independent predictors of ccRCC patients (Figure 5). Next, we figured up the AUCs for ROC curves of SRSM and clinical characters to test the accuracy of SRSM, and found that the AUCs of risk scores were 0.661, 0.683 and 0.719 for 1, 3 and 5 years, respectively (Figure 6). Finally, we normalized the number of SRSM points from 0 to 100, and plotted the sSR-LncRs expression level line between the total point axis and each prognostic axis (Figure 7). These results suggested that SRSM could be used as a risk factor for predicting the prognosis of ccRCC patients.

### LINC-PINT, AC108449.2 and AC007637.1 obtained prominent clinical relevance and significance

**Figure 8:**
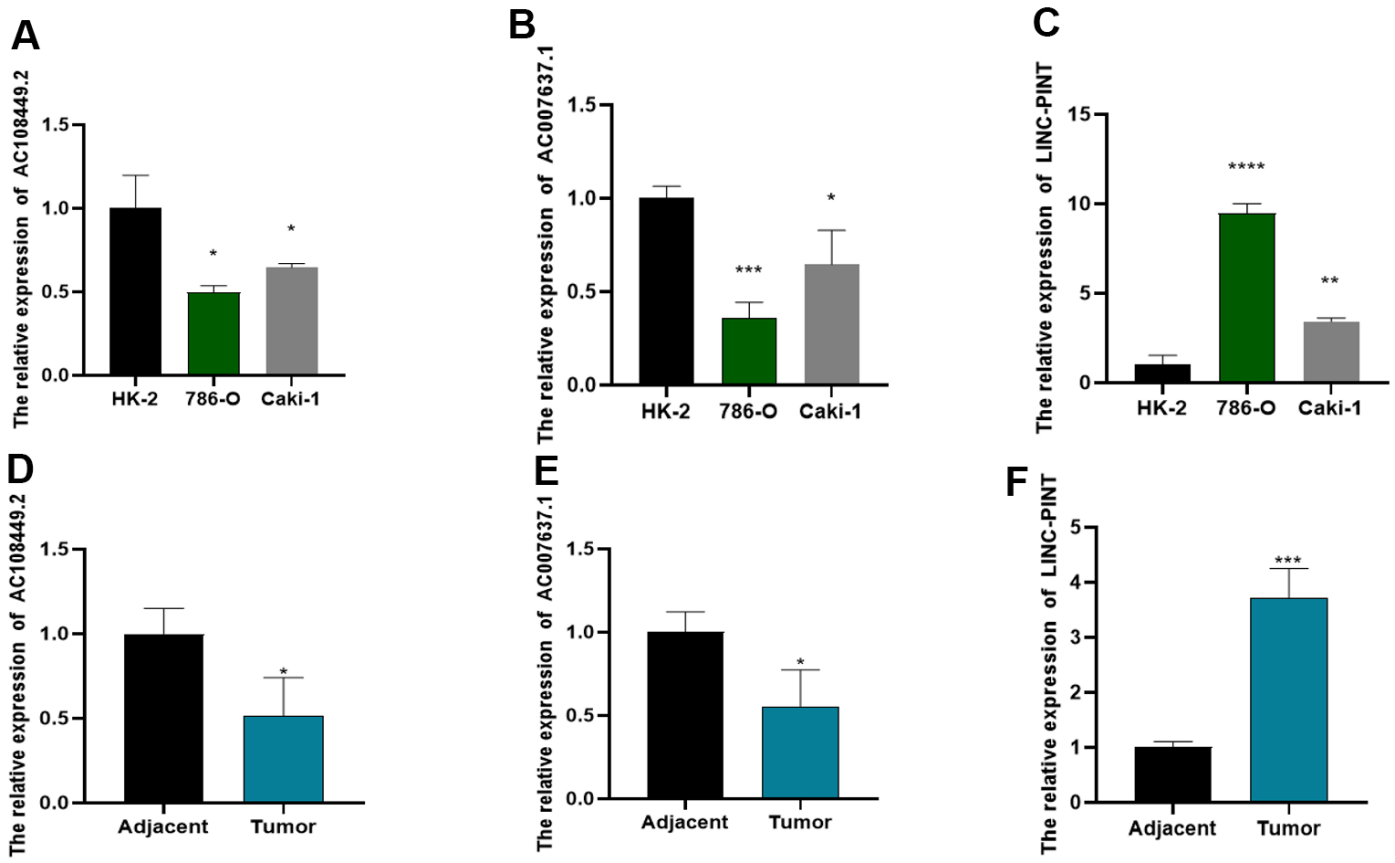
**The expression levels of LINC-PINT, AC108449.2 and AC007637.1 in cell lines and ccRCC clinical samples.** AC108449.2 (**A**) and AC007637.1 (**B**) expressed higher in HK-2 cell than that in ccRCC cell lines. The expression of LINC-PINT enhanced in ccRCC cell lines (**C**). The expression of AC108449.2 (**D**) and AC007637.1 (**E**) were increased in normal kidney tissues, while LINC-PINT (**F**) was up-regulated in ccRCC tissues.

Subsequently, we determined the expression of LINC-PINT, AC108449.2 and AC007637.1 in ccRCC tissues and cell lines. As shown in Figure 8A–8C, the expression level of LINC-PINT was significantly higher in tumor tissues than in adjacent normal tissues, but AC108449.2 and AC007637.1 were contrary. Similarly, the expression level of LINC-PINT in ccRCC cell line 786-O and Caki-1 was also higher than that in HK-2, but AC108449.2 and AC007637.1 were decreased in ccRCC cell lines. In addition, we found that LINC-PINT was highly expressed in the ccRCC tumor tissues, while the expressions of AC108449.2 and AC007637.1 were higher in adjacent normal tissues than that in ccRCC tissues (Figure 8D–8F).

### LINC-PINT, AC108449.2 and AC007637.1 were starvation-related and severely affected the invasion and migration abilities of ccRCC cells

**Figure 9:**
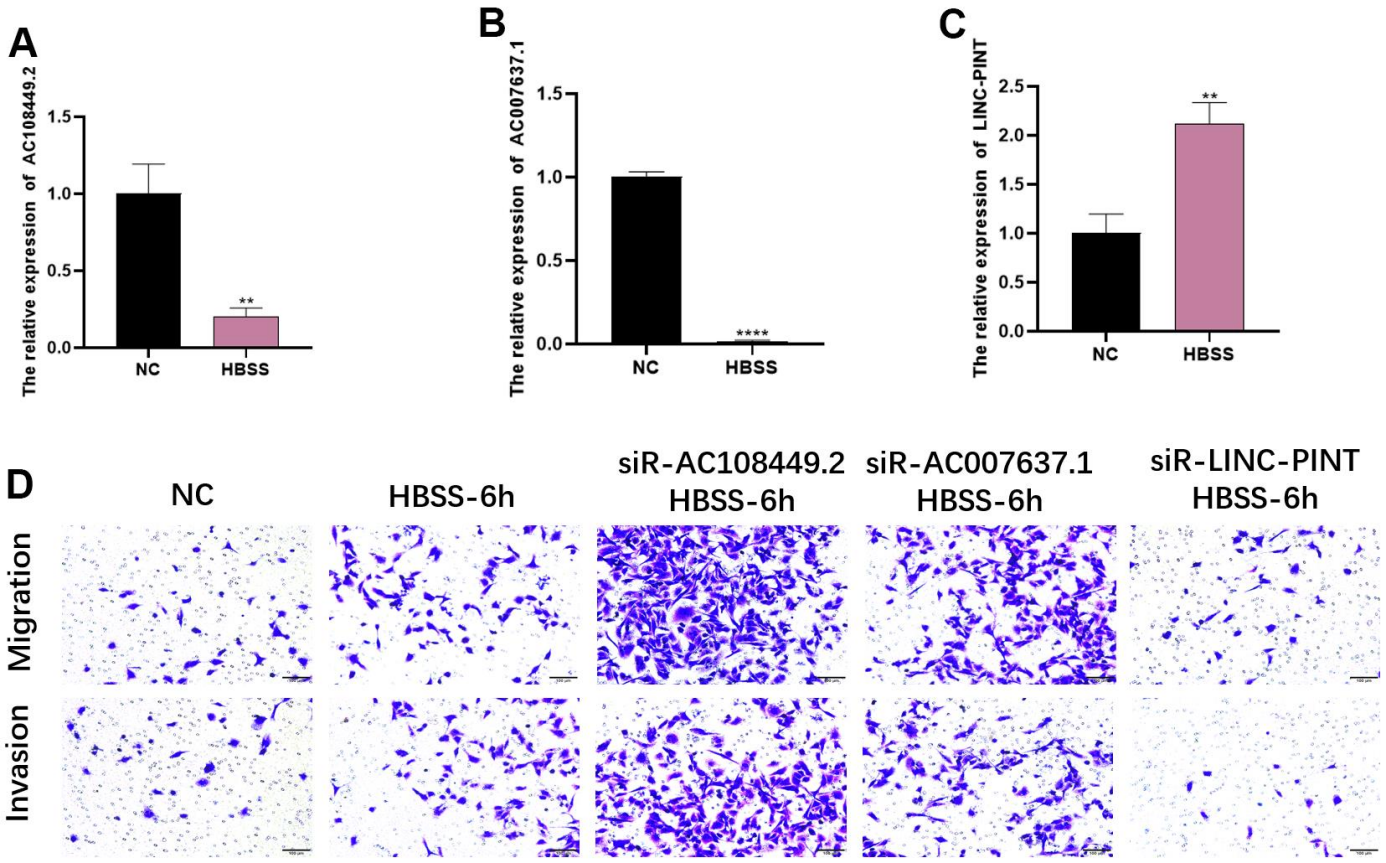
**The effects of LINC-PINT, AC108449.2 and AC007637.1 on invasion and migration in the starvation condition.** The expression levels of AC108449.2 and AC007637.1 (**A**, **B**) decreased in the starvation condition, but LINC-PINT (**C**) increased in that condition. The migration and invasion abilities of cells in starvation-condition were more robust than that in normal-condition. The knock-down of AC108449.2 and AC007637.1 could increase the starvation-induced migration and invasion ability of cells, while the deficiency of LINC-PINT significantly decreased those abilities induced by starvation (**D**).

In order to explore the role of LINC-PINT, AC108449.2 and AC007637.1 in the starvation-induced tumor microenvironment, we firstly established an HBSS-induced starvation model *in vitro* and detected the expression levels of LINC-PINT, AC108449.2 and AC007637.1. The results showed that LINC-PINT was significantly up-regulated under 6 hours starvation conditions, while AC108449.2 and AC007637.1 were further declined in 6 hours (Figure 9A–9C). To further verify whether LINC-PINT, AC108449.2 and AC007637.1 affected the metastasis ability of ccRCC, we first silenced LINC-PINT, AC108449.2 and AC007637.1 with siRNA in 786-O. We then conducted invasion and migration assays and noticed that starvation-induced 786-O cells obtained stronger invasion and migration abilities, however, silencing LINC-PINT attenuated this ability, while silencing AC108449.2 and AC007637.1 enhanced it (Figure 9D). Based on the aforesaid results, we can therefore deduce that in the starvation tumor microenvironment, LINC-PINT, AC108449.2 and AC007637.1 play a pivotal role in ccRCC metastasis.

## DISCUSSION

The invasion and migration of tumor cells seriously influence the survival of RCC patients [27]. Partial RCC patients have metastatic symptoms at the first diagnosis, and 20 -40 % of patients with localized lesions will eventually have metastasis [10, 28, 29]. Although the emergence of tyrosine kinase inhibitors (TKIs) has benefited patients with advanced ccRCC, distant metastasis is still a serious threat to patients, resulting in the rate of 10-year survival of less than 5% [30]. A series of evidence had demonstrated that lncRNAs play a crucial role in regulating the metastasis of ccRCC [26, 31]. In bladder cancer, LINC-PINT could inhibit the proliferation, invasion and migration by targeting miR-155-5p, and it was a potential prognostic marker for bladder cancer [32]. However, autophagy-related AC108449.2 was a risk factor in bladder cancer and could predict the prognosis of bladder cancer patients [33]. Exosomes secreted by ccRCC cancer stem cells (CSCs) promoted ccRCC cell proliferation and induced epithelial-mesenchymal transition [34]. In our research, we first proved that LINC-PINT was strongly associated with an increase in tumor cell invasion and migration, whereas AC108449.2 and AC007637.1 showed the opposite effect. Therefore, LINC-PINT, AC108449.2 and AC007637.1 have the potential to become new therapeutic targets for ccRCC.

Accumulating evidence has described many mechanisms for promoting cancer cells’ survival under nutrient-deprivation conditions (including starvation), which significantly affects the survival of cancer cells. Starvation-induced lncRNA AC020978 promoted the proliferation of non-small cell lung cancer through the PKM2 / HIF-1α axis, causing higher invasiveness [35]. P53-induced lncRNA TRINGS protected tumor cells from necrosis in the absence of glucose [36]. In the present study, we demonstrated for the first time that the starvation-induced tumor microenvironment significantly upregulated the expression of LINC-PINT and downregulated the expression of AC108449.2 and AC007637.1 in a time-dependent manner. In addition, these sSR-LncRs prominently regulated the invasion and migration of ccRCC.

Notwithstanding these findings demonstrate the role of SRSM in the prognosis evaluation of ccRCC patients and the close relationship between SR-LncRs (LINC-PINT, AC108449.2 and AC007637.1) and clinicopathological features. By establishing the SRSM, we can evaluate the potential risk of metastasis for patients before treatment and develop a personalized treatment plan based on the predicted results, leading to more accurate medical care. Furthermore, the SRSM can also be used for the early diagnosis of ccRCC, reducing disease incidence and mortality rates. In summary, the SRSM is an essential tool in clinical practice that can assist doctors in making more accurate diagnoses and treatment decisions, ultimately improving patients' quality of life. However, there are still some limitations, such as the feasibility of SRSM verified by a large number of clinical samples. With the continuous research about extracellular vesicles, it has been found that extracellular vesicles are of great significance in intercellular communication and participate in antigen presentation, cellular differentiation, proliferation, tumor immune response, and the ability of tumor cell migration and invasion. Therefore, it is still unknown whether LINC-PINT is rich in the extracellular vesicles secreted by ccRCC cells under starvation conditions, and thus affects other subcellular populations, which requires further exploration. Finally, the potential mechanism of starvation-induced lncRNA and the specific mechanism of LINC-PINT, AC108449.2 and AC007637.1 regulating the invasion and migration of ccRCC cells require establishing more of *in vitro* and *in vivo* models’ further exploration.

## CONCLUSIONS

In this study, we clarified the predictive effect of sSR-LncRs in the prognosis evaluation of ccRCC, and confirmed its clinical significance and metastasis correlation in ccRCC tissues and different ccRCC cell lines. LINC-PINT is over-expressed in tumor tissues and can further promote ccRCC metastasis under starvation conditions, while AC108449.2 and AC007637.1 show the opposite results. These findings not only establish an association between sSR-LncRs and ccRCC invasion and migration, but also provide an appropriate SRSM for ccRCC metastasis risk assessment.

## MATERIALS AND METHODS

### Clinical specimens

From February 2018 to October 2022, 50 ccRCC tumor and adjacent normal tissues were collected from patients who underwent tumor excision in the First Affiliated Hospital of Chongqing Medical University, and immediately stored in liquid nitrogen until RNA extraction.

### Cell cultivation and treatment

Normal human renal tubular epithelial cell HK-2 and ccRCC cell lines (786-O and Caki-1) were obtained from Procell (Wuhan, China). Cells were maintained in DMEM/F12 (HK-2), RPMI-1640 (786-O), McCoy's 5A (Caki-1) basal medium (Gibco, Gaithersburg, MD, USA) complemented with 10% fetal bovine serum (Biological Industries, Israel), 100 U/ml penicillin and 0.1 mg/ml streptomycin (Beyotime, Beijing, China) and maintained at 37° C in a humidified incubator under 5% CO2. 786-O cells were treated with Hank’s solution (Boster Biotechnology, China) for six hours to simulate tumor starvation environment, and then replaced with complete RPMI-1640 medium.

### Transwell assay

For the migration assay, 600μl RPMI-1640 medium containing 10% FBS was added into the lower chamber, and 4 × 10^5^ 786-O single-cell suspensions were inoculated into the upper chamber (Corning, NY, USA). For the invasion assay, Matrigel (diluted with RPMI-1640 basal medium to 1/9) was evenly spread over the upper chamber. Two hours later, 6 × 10^5^ 786-O cells in 100μl RPMI-1640 basal medium were added into the chamber with diluted Matrigel and placed on the lower chamber containing 600μl complete RPMI-1640 medium. After 24 and 48 hours incubating for the migration and invasion assays, the inserts were rinsed with PBS and fixated with 4% paraformaldehyde for 20 minutes. Finally, the inserts were dyed using 0.1% crystal violet solution for 20 minutes, and 5 fields (200X) of the insert were imaged by an optical microscope.

### Cell transfection

The siRNAs of LINC-PINT, AC108449.2 and AC007637.1 were transfected to knock down the expression of LINC-PINT, AC108449.2 and AC007637.1. The sequences used were: siR-LINC-PINT (sense:5′- CACUGUUGUCAGCAACAAAGC -3′, antisense: 5′- UUUGUUGCUGACAACAGUGAA -3′); siR-AC108449.2 (sense:5′- GGAAGAAGAUGGUGUUAUAGA -3′, antisense: 5′- UAUAACACCAUCUUCUUCCUU -3′); siR-AC007637.1 (sense:5′- GAGGAACAAGAGCAUUCAACA -3′, antisense: 5′- UUGAAUGCUCUUGUUCCUCUU -3′). For transient transfection, 5 × 10^5^ 786-O cells were cultivated in 6-cm dish. According to the manufacturer, when cells reached to 40-50% of the surface, cells were transfected with 10μl siRNAs (20uM) for 24 hours using 5μl Lipofectamine 3000 (Invitrogen, Invitrogen, Waltham, MA, USA), and RT-qPCR was used to verify the efficiencies of siRNAs.

### RNA isolation and RT-qPCR

Total RNA of cells and clinical tissues was extracted using TRIzol reagent (Abclonal, China). According to the manufacturer’s instructions, 1μg RNA was reverse transcribed with PrimeScript RT–qPCR kit (Abclonal, China). Quantitative PCR (qPCR) was performed with the SYBR(R) Prime-Script RT–PCR kit (Abclonal, China) using ABI 7500 real-time PCR system (Applied Biosystems, Waltham, MA, USA). The values of Ct were calculated using the 2-ΔΔCt method. The lncRNA values were standardized to the expression levels of β-actin. The primer sequences of lncRNAs and β-actin were shown in Table 1. All results were repeated three times.

**Table 1:** The primer sequences of LINC-PINT, AC108449.2, AC007637.1 and β-actin.

**LINC-PINT**	F primer (5’-3’)	GGTGTTTGCCTCTAGCCCTT
	R primer (5’-3’)	GGGCGGATGGCTTGAAATTG
**AC108449.2**	F primer (5’-3’)	GGCTTCTCGGATACAAGCCAA
	R primer (5’-3’)	GCCCTGCAAGATCACAAGAC
**AC007637.1**	F primer (5’-3’)	TTCCAGGCCACAAAGAGGAAC
	R primer (5’-3’)	AAAGAGAGGCTGCAAACGGAT
**β-actin**	F primer (5’-3’)	AAACGTGCTGCTGACCGAG
	R primer (5’-3’)	TAGCACAGCCTGGATAGCAAC

Note: F primer, forward primer; R primer, reverse primer.

### ccRCC transcriptome data from TCGA preprocessing

From The Cancer Genome Atlas (TCGA) data portal (https://portal.gdc.cancer.gov/), transcriptome RNA sequencing data of 539 ccRCC tumor tissues and 72 normal tissues were downloaded and extracted. Patients which OS≤30 days were excluded as they might die from unanticipated factors involving hemorrhage and infection. The original data of ccRCC patients were gathered for further evaluation. The Perl language script (http://www.perl.org/) was used to combine the transcriptomic RNA sequencing results and the clinical data of ccRCC patients into a matrix file.

### Acquiring the sSR-LncRs

SRGs were obtained from The Molecular Signatures Database version 4.0 (M16522 and M41835, http://www.broadinstitute.org/gsea/msigdb/index.jsp). Pearson correlation analysis was performed to determine the relationship between SRGs and the expression level of lncRNAs in ccRCC patients. SR-LncRs were screened using the criteria of | r | > 0.7 and P < 0.001. In addition, sSR-LncRs were filtered through univariate COX analysis and survival package of R software (P < 0.001). Finally, the sSR-LncRs were divided into the harmful or protective parts using hazard ratio (HR).

### Starvation-related risk score model (SRSM)

SRSM was constructed based on the selected sSR-LncRs through multivariate COX regression analysis. The risk score of ccRCC patients was computed by multiplying the expression level of the sSR-LncRs by the coefficients of the Cox regression analysis. The formula was as followed, [(Expression level of AC009120.2) * (-0.5517)] + [(Expression level of AC108449.2) * (-0.4033)] + [(Expression level of AC007637.1) * (-0.4986)] + [(Expression level of AC008870.2) * (0.9801)] + [(Expression level of LINC-PINT) * (0.4225)]. Patients with ccRCC were divided into high-risk and low-risk groups on the basis of the median score.

### Bioinformatics analysis

The survival package was used to evaluate the survival rate of patients in SRSM. The survival ROC package was used to generate and calculate the receiver operating characteristic (ROC) curves and area under the curve (AUC) to evaluate the accuracy of SRSM. Multivariate Cox regression analysis was performed to validate the independent prognostic predictors of ccRCC patients. The nomogram was applied to forecast the survival rate of ccRCC patients using the rms package.

### Statistical analysis

The SPSS version 27.0 (SPSS, Chicago, IL, USA) and GraphPad Prism 8.0 (GraphPad Software Inc, La Jolla, CA) were used for statistical analysis. Data are presented as means ± SD. Fisher's exact test was utilized for estimating the correlation between the expression level of sSR-LncRs and clinicopathological features. Student's T-test, ANOVA and post-hoc test were used to compare differences among two or more groups. Statistical significance was defined as *P* < 0.05.

### Availability of data and materials

Authors can provide all data sets analyzed during the study on reasonable requirements.
